# Population pharmacokinetics and safety of continuous oxacillin in preterm and term neonates and infants

**DOI:** 10.1128/aac.01777-25

**Published:** 2026-04-20

**Authors:** Adam Lee, Catherine Liu, M. Tuan Tran, Suna Phal, Charles A. Peloquin, Delma Nieves, Edmund Capparelli, Antonio C. Arrieta

**Affiliations:** 1 Department of Infectious Diseases, Children's Hospital of Orange County 20209, Orange, California, USA; 2 Skaggs School of Pharmacy and Pharmaceutical Sciences, University of California-San Diego 8784, La Jolla, California, USA; 3 Department of Pharmacy, Children's Hospital of Orange County 20209, Orange, California, USA; 4 Department of Translational Research, Infectious Disease Pharmacokinetic Laboratory, University of Florida 3463, Gainesville, Florida, USA; 5 Department of Pediatrics, University of California-Irvine 8788, Irvine, California, USA; University Children's Hospital Münster, Münster, Germany

**Keywords:** pharmacodynamics, pharmacokinetics, sepsis, neonatal

## Abstract

*Staphylococcus aureus* increasingly causes late-onset neonatal sepsis with mortality ≤25%. Oxacillin treats methicillin-susceptible *Staphylococcus aureus* (MSSA). No previous studies inform dosing. Time above MIC of unbound oxacillin (*f*T>MIC) 100% of dosing interval is a desired pharmacodynamic (PD) target in immunocompromised/septic patients. Our objective was to characterize oxacillin pharmacokinetics (PK) and PD in young infants. We conducted a prospective PK/PD study in infants ≤90 days. We divided four cohorts per gestational (GA <32/≥32 weeks) and postnatal age (PNA <14/≥14 days). Patients received 25 mg/kg loading doses followed by continuous infusions of 120 (<32 weeks/<14 days) or 160 mg/kg/day. Population PK parameters were estimated using NONMEM. Dosing regimens were evaluated using Monte Carlo simulations to estimate the probability of PD target attainment. Outcomes were collected. We enrolled 22 infants, median (IQR) GA 38 weeks (30–39) and PNA 32 days (12–46). PNA and weight were covariates for oxacillin clearance. Unbound steady-state oxacillin concentrations remained >MIC 0.5 throughout the dosing interval for >90% of modeled patients for all age groups on 160 mg/kg/day continuous infusions. Infants with PNA ≤28 days met PD targets at MIC 1; <90% of all modeled patients on continuous infusion met PD targets at MIC 2. Modeled infants receiving 200 mg/kg/day oxacillin divided q4h/q6h failed to meet PD targets up to MIC 2. All patients survived to discharge. A few serious adverse events were noted. 160 mg/kg/day continuous oxacillin was well tolerated and achieved PD targets for MIC ≤0.5 across all age groups. Modeled intermittent dosing ≤200 mg/kg/day divided q4h/q6h, 8 times the Food and Drug Administration-labeled dose of 25 mg/kg daily, failed to achieve PD targets. The FDA-recommended oxacillin dose is inadequate and may help explain high mortality.

## INTRODUCTION

Oxacillin is a standard of care semi-synthetic penicillin for methicillin-susceptible *Staphylococcus aureus* (MSSA) infections. *Staphylococcus aureus* (*S. aureus*) has increasingly been reported as the cause of neonatal late-onset sepsis and is associated with a high mortality that approaches 12%–25% in premature infants, who disproportionately comprise the majority of the burden of infection and mortality. Of those, 40% die within 72 hours from the onset of infection (1–8). Prompt effective therapy is crucial to improve outcomes. To date, no studies exist to inform oxacillin dosing recommendations in preterm and term newborns and infants under 90 days of age.

Oxacillin is a time-dependent beta-lactam antibiotic with an estimated protein binding of 90% (9). Clinical success is tied to the proportion of time during the dosing interval that unbound oxacillin remains above the minimum inhibitory concentration (*f*T > MIC). Literature suggests that the desired pharmacodynamic (PD) target associated with clinical success for immunocompromised or critically ill hosts is *f*T > MIC of 80%–100%(9–11). Clinicians caring for neonates with severe infections may find greater success in achieving *f*T 100% > MIC with continuous infusions, rather than intermittent (12).

The current recommended dose of oxacillin in neonates on the United States Food and Drug Administration (FDA) label is 25 mg/kg/day (13). In contrast, for severe infections such as infectious endocarditis, the American Heart Association recommends oxacillin dosing of 200 mg/kg/day divided every 4–6 hours (q4h to q6h, respectively) for children (excluding neonates and infants) (14). However, no prior PK/PD studies exist to guide dosing recommendations in premature or term newborns and infants up to 90 days of age.

Due to the discrepancy in these dosing recommendations and the lack of high-quality literature on oxacillin in this age group, we felt it was crucial to better characterize the pharmacokinetics (PK) of oxacillin to inform dosing recommendations. Population pharmacokinetic data from preterm and term infants between 3 and 90 days of age on continuous infusion were utilized to simulate the oxacillin probability of target attainment (PTA) with different dosing regimens. A loading dose of oxacillin was given prior to continuous infusion. We preferred treating using this loading dose-continuous infusion regimen given that others have demonstrated a greater likelihood than intermittent infusions in achieving *f*T 100% > MIC (9, 12, 15). We hypothesized that the unbound oxacillin serum concentration, when dosed at 160 mg/kg/day administered as continuous infusions, would remain above the Clinical and Laboratory Standards Institute (CLSI) breakpoint MIC of 2 mcg/mL throughout the entire dosing exposure (as a surrogate for *f*T 100% > MIC). We also evaluated whether virtual regimens of intermittent oxacillin dosed at 200 mg/kg/day divided q4h would achieve 90% PTA of *f*T 100% > MIC of 2 mcg/mL.

## MATERIALS AND METHODS

### Study design

This was a prospective, phase 1, open-label, single-center PK study of oxacillin in infants. Inclusion criteria included the following: postnatal age (PNA) > 3 to ≤ 90 days; appropriate vascular access for oxacillin administration; likely survival > 72 hours after enrollment; and receiving oxacillin per standard of care for suspected or confirmed *S. aureus* infection. A protocol amendment enabled enrollment for patients receiving oxacillin as perioperative prophylaxis, due to slow enrollment. Participants were excluded for the following conditions: undergoing or receiving therapeutic hypothermia within the previous 24 hours; renal dysfunction defined as undergoing dialysis, or urine output < 0.5 mL/kg/h, or serum creatinine > 1.7 mg/dL; liver enzymes > 5 times the upper limit of normal; receiving extracorporeal membrane oxygenation (ECMO) treatment; or any condition which would make the participant, in the opinion of the investigators, unsuitable for the study. The Institutional Review Board approved this study. Study participants were enrolled after obtaining written informed consent from the parent or legal guardian.

The following laboratory values were collected within 24 hours of oxacillin initiation and throughout the clinical course as per standard of care: total white blood cell count, hemoglobin, platelets, absolute neutrophil count, serum creatinine, alanine aminotransferase (ALT), aspartate aminotransferase (AST), albumin, bilirubin (total and direct), and alkaline phosphatase.

**Fig 1:**
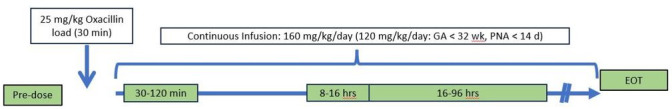
Dosing schematic. White boxes: dosing schedule, with continuous infusion (160 or 120 mg/kg/day depending on gestational/chronological age) started immediately after oxacillin loading dose (25 mg/kg). Green boxes: time windows for blood draws for sampling of plasma oxacillin levels. EOT: end of treatment PK sample.

Patients were stratified into one of four age cohorts based on gestational age (GA, <32 weeks and ≥32 weeks) at birth and PNA (<14 days and ≥14 days) at the time of study entry, with a goal of 6 patients per cohort for a total of 24 patients. This goal sample size estimation was made per the recommendations of expert pharmacometricians; no power analysis was done. Given a lack of literature on oxacillin pharmacokinetics, the gestational age cutoff for each cohort was arbitrarily chosen based on a similar stratification in a separate PK study on meropenem (16). The cutoff for postnatal age of 14 days was chosen as renal clearance drastically changes during the first 14 days of life, which was felt to likely affect oxacillin clearance (17). All patients received a single loading dose of 25 mg/kg oxacillin given over 30 minutes. This was immediately followed by a continuous infusion of oxacillin at 120 mg/kg/day for patients in Group 1 (GA <32 weeks and <14 days); all other patients in Groups 2–4 (GA <32 weeks and ≥14 days, GA >32 weeks and <14 days, GA >32 weeks and ≥14 days, respectively) received continuous infusions of oxacillin at 160 mg/kg/day. Table 1 and Fig. 1 summarize cohort stratification and dosing schematic. The rationale for selecting a dose of 160 mg/kg/day was consistent with the local standard of care, as recommended by the Infectious Diseases team. Our typical dosing recommendation at our institution is between 150 and 200 mg/kg/day as intermittent infusions, within the range recommended for serious MSSA infections in older pediatric populations (18, 19, 20). However, generalizing from literature on other antimicrobials that continuous infusions maximize time above MIC better than intermittent infusions, and with a scarcity of literature to support oxacillin dosing recommendations in this age group, we opted to reduce over oxacillin exposure to minimize chances of toxicity via a continuous infusion of 160 mg/kg/day (12). 120 mg/kg/day dosing for patients in Group 1 was chosen to minimize potential toxicity, since renal immaturity in this sub-population would be expected to result in higher concentrations than in other infant groups with dosing of 160 mg/kg/day. The rationale for the dosing schedule of loading dose, followed by continuous infusion, was to rapidly achieve a high serum concentration after the loading dose and to remain at steady state while on continuous infusion. This facilitated convenience sampling of blood for PK evaluation with other timed blood draws per standard of care.

**TABLE 1:** Patient demographics^*a*^

Demographics and characteristics of patients	Cohort 1 GA ≥ 32 weeks PNA 14–90 days	Cohort 2 GA ≥ 32 weeks PNA < 14 days	Cohort 3 GA < 32 weeks PNA 14–90 days	Cohort 4 GA < 32 weeks PNA < 14 days	Total
*N*	10	5	6	1	22
Gestational age (weeks)	39 (38–39)	39 (38–39)	29 (26–30)	26	38 (30–39)
Postnatal age (days)	32 (23–40)	7 (4–11)	66 (40–81)	5	32 (12–46)
Male, *N* (%)	8 (80)	3 (60)	4 (67)	1 (100)	15 (68)
Staphylococcal infections, *N*	7	1^*b*^	2	0	10^*b*^
Perioperative prophylaxis, *N*	1	0	0	0	1
Empiric antibiotics, *N*	2	4	4	1	11
Serum albumin (g/dL)	3.4 (3.3–3.8)	3.1 (3.0–3.5)	3.1 (2.7–3.2)	3.2	3.2 (3.0–3.6)
Serum creatinine (mg/dL)	0.25 (0.2–0.3)	0.43 (0.3–0.5)	0.25 (0.2–0.33)	1.2	0.3 (0.2–0.36)

^*a*^ All data are median (IQR) unless otherwise stated. GA, gestational age; PNA, postnatal age; IQR, interquartile range.

^*b*^ One patient treated for methicillin-susceptible *S. epidermidis* infection. All other infections were methicillin-susceptible *S. aureus*.

The duration of oxacillin was determined by the treating physician teams; if a patient was placed on oxacillin for coverage of *S. aureus* for possible late-onset sepsis, and sepsis was ruled out by 36–48 hours, oxacillin and any other antimicrobials would be discontinued as standard of care. If *S. aureus* infection was confirmed, then oxacillin would be continued as long as deemed necessary for adequate treatment of the infection. Administration of other antimicrobial agents was also allowed per standard of care. Interruptions to the continuous oxacillin infusion according to medication incompatibilities were allowed and minimized when possible. Appropriate blood, urine, and/or cerebrospinal (CSF) cultures were obtained per standard of care.

PK sampling (minimum 25 μL per sample) was collected on all infants via heel stick, peripheral stick, or from available arterial/venous lines after an appropriate volume of blood waste was aspirated, to avoid erroneous oxacillin detection from the infusing catheter. A maximum of five blood samples was obtained per patient. A baseline sample (before 25 mg/kg oxacillin loading dose if patients received oxacillin prior to enrollment) was obtained, and at the following time points after initiation of continuous infusion: 30–120 minutes, 8–16 hours, 16–96 hours, and within 1 hour after the end of infusion. A sparse sampling procedure was employed for patients in Group 1, where only a maximum of three blood samples would be obtained at baseline, 30–120 minutes, and 16–96 hours, to minimize total blood volume taken in this age cohort. Four additional patients outside of Group 1 also had sparse sampling due to physician concerns of excessive phlebotomy. Because the oxacillin was dosed as a continuous infusion, it was presumed they would be at steady state by no longer than 16 hours (given a half-life ranging from 1.2 to 3 hours according to GA and PNA) (21). The wide sampling times were selected to facilitate the collection of PK samples with other standard-of-care blood draws. If CSF was obtained as per standard of care to rule out meningitis, any additional CSF (minimum of 25 μL) was scavenged with permission of the treating physician teams for PK analysis.

The quantification of oxacillin plasma and CSF concentrations was done at the Infectious Disease Pharmacokinetics Laboratory at the University of Florida using validated liquid chromatography with tandem mass spectrometry assays (LC-MS/MS). The calibration range was 2–100 mcg/mL. Across the range of calibrators, all percent coefficient of variation (%CV) for within-day and between-day assay precision were less than 10%, and all values for within-day and between-day accuracy were between 90% and 100% of the expected values.

Samples were stored at −80°C until the time of analysis. Oxacillin was stable in plasma for 1 year at −80°C. Long-term stability was tested within the Infectious Diseases Pharmacokinetics Laboratory. Quality control samples (QCs) were tested at 1 year and 2 years after they were made and ranged. At 2 years, all QCs were within 88%–100% of their nominal values, well within the range of variability. The average time between sample collection and analysis was about 6 months.

The study was conducted in accordance with the Declaration of Helsinki, Good Clinical Practice requirements.

### Safety assessments

Safety assessments, up to 14 days after the last oxacillin exposure on study, included adverse event (AE) monitoring, physical examinations, ophthalmological evaluations, and laboratory and imaging tests performed before, during, and after oxacillin administration. Laboratory tests were monitored at least weekly if collected as part of standard of care, though laboratory tests were not collected specifically for study purposes. All AEs were monitored until resolution or stabilization. Specific safety endpoints of interest included evolution of neutropenia <1,500 cells/μL, aspartate aminotransferase (AST) increase of 1.25-fold above normal, alanine aminotransferase (ALT) increase of 1.25-fold above normal, direct bilirubin elevation, total bilirubin elevation, alkaline phosphatase elevation, doubling of serum creatinine from baseline, and need for red cell transfusion as consequence of study-related phlebotomy.

### Pharmacokinetic model

PK modeling was done using non-linear mixed effect modeling (NONMEM 7.5.0, Icon, Dublin, Ireland) with the first-order conditional estimation method with interaction (FOCE-I). A one-compartment model with first-order elimination (ADVAN1 TRANS2) was chosen to fit the data. Clearance (Cl) was allometrically scaled by weight (WT_Kg_) and included before assessment of other covariates. Inter-individual deviations from typical population parameters were assumed to follow a log-normal distribution. A combined additive and proportional within-subject error model was chosen to characterize the residual error. We investigated the relationship between the base population PK model and the following possible covariates: GA, PNA, postmenstrual age, height (cm), body surface area, albumin, serum creatinine, and baseline serum transaminases. A covariate was considered significant and retained in the final model if its inclusion dropped the objective function value (OFV) by more than 3.84 (corresponding to *P*-value < 0.05), and was also significant based on the bootstrap 95% confidence intervals of the covariance effect.

Goodness-of-fit plots were generated using R version 4.4.2 (https://www.r-project.org) to assess the appropriateness of the final model. Model reliability was evaluated using 1,000-set bootstrapping procedures and the program Wings for NONMEM (https://wfn.sourceforge.net).

### Simulations and pharmacodynamic assessments

Using R, 10,000 virtual patients were simulated to receive modeled continuous and intermittent infusions of oxacillin. Continuous infusions were evaluated at 160 mg/kg/day. Exposure was evaluated for the PD target of steady state unbound concentration remaining above MIC throughout continuous dosing exposure (as a surrogate for *f*T 100% > MIC) for a range of MIC’s ≤0.25 to 2 mcg/mL. Simulated intermittent infusions were evaluated using dosing according to references commonly used for *Staphylococcus aureus* infections: Lexi-drug and *Infectious Endocarditis in Childhood: 2015 Update* (14, 22). Intermittent dosing regimens evaluated were 100 mg/kg/day divided q6h, 200 mg/kg/day divided q4h, and 200 mg/kg/day divided q6h. For modeled intermittent oxacillin infusions, exposure was evaluated to the following PD targets for efficacy: *f*T > MIC 80% and 100% (free plasma oxacillin concentrations above MIC for 80% and 100% of dosing interval, respectively) at a range of MICs ≤0.25 to 2 mcg/mL. Free-drug concentrations of oxacillin were estimated at 10% of total concentrations as reported in the literature (9). A Shiny app was constructed from the final population PK model, which allows users (after inputting parameters for age, weight, total daily dose, and dose interval) to dynamically assess concentration profiles, time above MIC, and achievement of PD targets, and is accessible at https://catwliu.shinyapps.io/oxacillin_neonate_poppk/.

### Statistical analysis

Continuous variables were summarized as the number of observations, mean, standard deviation, median, and range (maximum and minimum) values. Categorical variables were summarized using frequency count and percentages. No statistical analysis was performed on adverse events related to laboratory values, as these were recorded only if laboratory values were obtained as part of standard of care.

## RESULTS

Twenty-nine patients consented to participate. Six were excluded before the initiation of study procedures for varying reasons (withdrawal of consent, evolution of clinical instability). One was unevaluable because oxacillin was discontinued before PK sampling. Thus, 22 enrolled individuals completed study procedures. Eleven received empiric oxacillin for suspected infection, 1 received oxacillin for perioperative prophylaxis, 9 had confirmed *S. aureus* bacterial infections with osteomyelitis (*n* = 1), pneumonia (*n* = 3), bacteremia (*n* = 3), and skin/soft tissue infection (*n* = 2), and 1 had oxacillin-susceptible *S. epidermidis* infection (bacteremia). Table 1 summarizes patient demographics.

From 22 patients, 89 plasma and 1 CSF samples were obtained. Ten plasma samples had concentrations below the lower limit of quantification (nine were baseline samples) and were excluded to avoid biasing model development. Five patients (23%) had only 2 PK samples obtained (after initiation of oxacillin continuous infusion); all others had ≥ 3 PK samples obtained. The median total oxacillin concentration was 20.2 mcg/mL (IQR 11.8–36.2). The singular CSF concentration was not included in the final PK model.

### Pharmacokinetic model

The final model was a one-compartment population PK model with first-order elimination:

CL (L/h) = 1.01 * (WT_Kg_/3.4)^0.75^ * (PNA_days_/36)^0.433^

Vd (L) = 1.87 * WT_Kg_/3.4

**Fig 2:**
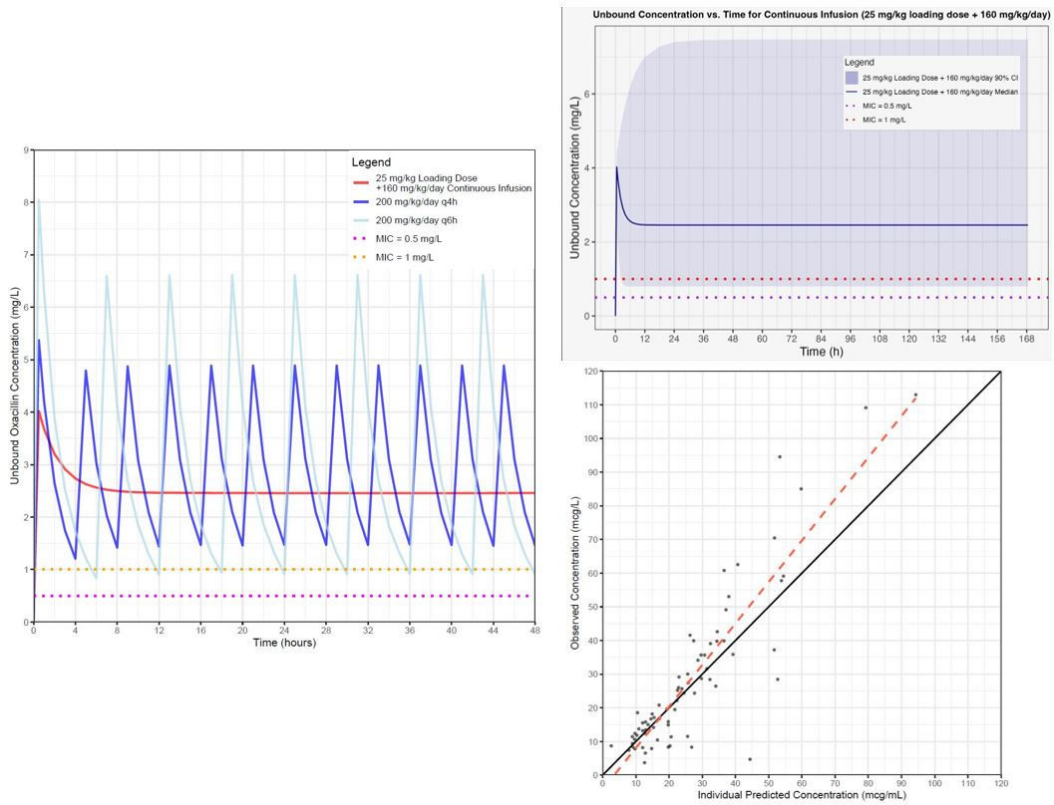
Left: Time-concentration curve of oxacillin (median concentrations depicted) during the first 48 hours of administration. Note that the loading dose enables free oxacillin concentrations to remain above MIC during the early hours after treatment initiation. Top right: Time-concentration curve of median oxacillin concentrations with shaded 90% confidence intervals, MIC 0.5 and 1 mg/L shown for comparison. Bottom right: A goodness-of-fit plot of individual predicted vs observed concentrations.

PNA_days_ (postnatal age in days) was a significant covariate for CL, associated with a 2.2-fold increase in CL (weight adjusted) from 14 to 90 days of age. Table 2 shows the typical values for CL and Vd. Between-subject variability (BSV) could only be estimated for CL. η-shrinkage for CL was low (8.27%). Final model estimate values for covariates, individual variances, and 95% confidence intervals are listed in Table 2. The model was considered reliable as the model estimates were within the bootstrapping 95% confidence intervals. The goodness-of-fit plot demonstrated that the model predictions generally fit the observed concentrations (see Fig. 2). A projected time-concentration curve of oxacillin (median concentrations depicted) with varying dose regimens is also shown, as well as a time-concentration curve of oxacillin administered as a continuous infusion with preceding loading dose for a typical infant 36 days postnatal age and weighing 3.4 kg (median and 90% confidence intervals shown).

**TABLE 2:** Final pharmacokinetic model parameters^*a*^^,^^*b*^^,^^*c*^

			Bootstrapping 95% confidence interval		
Parameter	Final estimate	Shrinkage (%)	Lower bound	Median	Upper bound
CL (Θ1)	1.01	–	0.791	1.01	1.3
V (Θ2)	1.87	–	1.07	1.78	2.61
Effect of PNA on CL (Θ3)	0.433	–	0.133	0.422	0.709
IIV CL (hCL)	0.568	8.27	0.373	0.54	0.683

^*a*^ CL, clearance; V, volume of distribution; PNA, postnatal age in days; IIV, inter-individual variability.

^*b*^ Final Model: CL (L/h) = 1.01 * (WT_Kg_/3.4) 0.75 * (PNA_days_/36) 0.433 Vd (L) = 1.87 * WT_Kg_/3.4.

^*c*^ –, lack of a value.

### Probability of achieving targeted pharmacodynamic exposures

**Fig 3:**
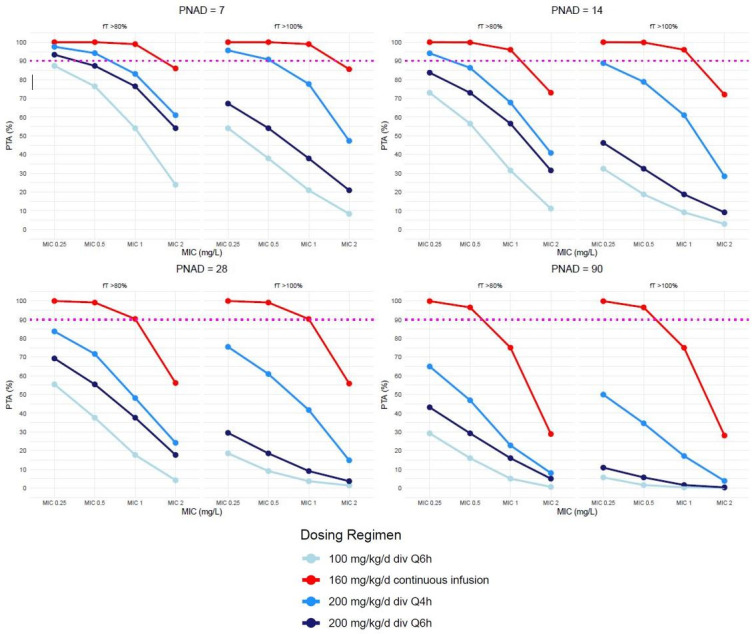
Probability of PD target attainment (PTA): Simulations of continuous and intermittent infusions of oxacillin. PNAD: Post-natal age in days. Dashed lines represent 90% probability of target attainment for separate PD targets of *f*T 80% > MIC (left of each graph) or *f*T 100% > MIC (right of each graph).

Simulations for continuous and intermittent infusions at various doses are displayed together in Fig. 3. PD targets of *f*T 80% > MIC are also shown for comparison. For continuous infusions (simulated dosing of 160 mg/kg/day), steady-state concentrations were chosen as a surrogate marker for *f*T 80 and 100% > MIC.

Patients at all evaluated chronological ages (PNAD 7, 14, 28, and 90) on continuous infusions met steady-state concentrations above MIC 0.5 mcg/mL. Patients 28 days of age or younger achieved *f*T 100% >MIC at an MIC of 1. Continuous oxacillin at 160 mg/kg/day was not adequate to meet pre-specified pharmacodynamic targets for all postnatal ages at the CLSI breakpoint MIC of 2.

Patients with a chronological age of 7 days’ postnatal age on intermittent infusions of 200 mg/kg/day divided q4h achieved the pre-specified PD target of *f*T 100% >MIC up to MIC 0.5. However, no other age group (PNAD 14, 28, and 90) on intermittent infusions achieved this pre-specified PD target at any MIC. No patient, on continuous or intermittent infusions, achieved the PD target at the CLSI breakpoint MIC of 2.

### Patient outcomes

All 10 patients with microbiologically defined infections (9 *S. aureus*, 1 oxacillin-susceptible *S. epidermidis*) had MIC to oxacillin ranging 0.25–1 mcg/mL (9 of 10 isolates had MIC testing). Each patient received continuous infusion as per protocol. The majority of measured oxacillin concentrations while on continuous infusion (35 of 36 measured concentrations in this group of patients, and 26 of 27 steady-state concentrations) remained above MIC for the respective pathogen. Each patient was successfully treated with complete resolution of infection.

### Safety

There were 45 adverse events (AEs) and 3 serious adverse events (SAEs) in total. These are summarized in Table 3. Eighteen patients had at least 1 AE. Twelve AEs were possibly related to oxacillin administration, including transient neutropenia and thrombocytopenia (*n* = 1, during 5-week course of oxacillin for osteomyelitis), elevated AST (*n* = 2), elevated ALT (*n* = 1), elevated alkaline phosphatase (*n* = 1), elevated direct bilirubin (*n* = 2), doubling of serum creatinine (*n* = 2), fever (*n* = 1), and rash (*n* = 1). The two patients with doubling of serum creatinine were both on concomitant nephrotoxic medications (one patient was on vancomycin, furosemide, and ibuprofen; the other patient was on furosemide drip and acyclovir) as likely contributors to kidney injury; each patient’s serum creatinine later normalized. One infant discontinued oxacillin early due to transient liver injury (AST 300 and ALT 141, without cholestasis) that was possibly related to oxacillin, though also had symptomatic rhinovirus/enterovirus infection.

**TABLE 3:** Overall safety summary^*a*^

Treatment-emergent and drug-related adverse events		N
Patients with at least 1 AE		18
Patients with at least 1 SAE		3
Total number of AEs		45
	Possibly related	12
	Unrelated	33
Total number of SAEs		3
	Possibly related	0
	Unrelated	3
Deaths		0
Laboratory value AE		
	AST	2
	ALT	1
	Alkaline phosphatase	1
	Total bilirubin	0
	Direct bilirubin	2
	Serum creatinine	2
	ANC < 1,500	1
	Thrombocytopenia	1
ROP		3
NEC		0
IVH		0
Patients with ≥ 1 A/B/D		13
Reintubation		2
Fever		4
Rash		1
Red blood cell transfusion		2
AE leading to study drug discontinuation		1

^*a*^ AE, adverse event; SAE, serious adverse event; AST, aspartate aminotransferase; ALT, alanine aminotransferase; ANC, absolute neutrophil count; ROP, retinopathy of prematurity; NEC, necrotizing enterocolitis; IVH, intraventricular hemorrhage; A/B/D, apnea, bradycardia, desaturation spells.

Other AEs included apnea/bradycardia/desaturation spells (*n* = 13), fever (*n* = 3), anemia (*n* = 9), and retinopathy of prematurity (ROP, *n* = 3). Of the nine patients with anemia, two required transfusions prompted by a combination of frequent standard of care and study-related blood draws for PK analysis. Of three patients with ROP, two had pre-existing ROP that remained stable, while one had pre-existing ROP that progressed on oxacillin therapy.

Three patients had SAEs (one each). None of them was related to oxacillin administration. These included re-intubation for respiratory failure (post-extubation atelectasis, *n* = 2) and development of junctional bradycardia requiring transcutaneous pacing (in one patient in the immediate postoperative period with surgically corrected D-transposition of the great arteries). No patients died.

## DISCUSSION

Literature on optimal oxacillin dosing in premature and term neonates and infants is very limited. There are no prior pediatric pharmacokinetic studies on oxacillin. Instead, prior studies on children, dated 1963 to 1978, mainly focused on safety and clinical efficacy (many subjects with skin/soft tissue infections) (21, 23–26). These studies included children whose ages ranged from premature infants (of unclear gestational and chronological ages) to toddlers, receiving oxacillin with doses ranging from 25 to 400 mg/kg/day. The available data showed that the serum half-life of oxacillin decreased from 1.6 hours in the second week of life to 1.2 hours in the third week of life. This may be explained by the significant changes in renal maturation exhibited in the neonatal period, demonstrating increases in creatinine clearance, which has been shown for other renally cleared antimicrobial agents (27, 17, 28). However, as this prior literature focused on patients receiving intramuscular oxacillin, it can be difficult to extrapolate intravenous pharmacokinetics from these studies. Furthermore, infants demonstrated no accumulation of oxacillin by 6 hours post-dose despite receiving between 200 and 400 mg/kg/day as intramuscular injections, suggesting an inability to achieve stringent PD targets of *f*T 100% > MIC even with high-dose intermittent oxacillin (26). Altogether, a dosing regimen meeting this desired target in this age group has not been harmonized yet, which is concerning given a mortality as high as 25% in *S. aureus* late-onset sepsis.

This is the first known PK/PD study of oxacillin in premature and term neonates and young infants up to 90 days of age. Our data suggest highly concerning findings that throughout this entire age group, oxacillin is incapable of achieving stringent PD targets at the CLSI breakpoint MIC of 2 mcg/ml for immunocompromised hosts, even at doses much higher than currently recommended by the FDA. Though outside the scope of this study, the inability of oxacillin to achieve stringent PD targets at these doses may be a contributing factor to the high mortality observed with severe *S. aureus* neonatal late-onset sepsis (1–3).

We did find 160 mg/kg/day continuous oxacillin infusions to be safe in premature and term infants. Assuming oxacillin was 90% protein bound, based on literature estimates, we also reached the desired PD target of 100% *f*T > MIC up to an MIC of 0.5 (coinciding with our local institutional MIC_90_ of MSSA) up to 90 days of postnatal age, and up to an MIC of 1 for infants up to 28 days of postnatal age. Thus, not all infants throughout this age group could achieve the desired PD targets at an MIC of 1. Furthermore, intermittent infusions of oxacillin at 200 mg/kg/day divided q4h were able to achieve the PD target for an MIC of 0.5 in patients up to 7 days’ postnatal age, but not in older children up to 90 days of postnatal age. Before 7 days of postnatal age, neonates have significant renal immaturity with much slower oxacillin clearance than older infants, which would enable a greater probability of target attainment. However, with increasing age, renal maturation, and clearance, infants beyond the first week of life on intermittent infusions have diminishing ability to achieve desired PD targets associated with clinical success at any MIC. This suggests that oxacillin should be administered as continuous infusions, not intermittent infusions, in infants with late-onset sepsis due to *S. aureus* with an MIC up to 0.5 mcg/mL; for infants up to 28 days PNA, continuous infusions can be used up to MIC 1. For infections with an *S. aureus* isolate with an MIC at the current breakpoint of 2 mcg/mL, an alternative agent should be considered. This also calls into question whether the current breakpoint should be reevaluated, particularly for severe infections in immunocompromised patients. Clinicians who rely on oxacillin should review their local antibiogram to determine their institution’s MIC_90_ to evaluate if oxacillin is the appropriate agent for *S. aureus*.

From our data, we found that the population PK model fit the observed data reasonably well. The model estimates for CL and Vd were very close to the bootstrap median values, and the 95% confidence intervals were narrow. Furthermore, by including a broad range of gestational and chronological ages, we were able to account for some post-birth maturational changes that affect the clearance of oxacillin. We found increasing age (PNA_days_) to be a significant covariate affecting oxacillin clearance, which is consistent with research on other antimicrobials where profound age-related renal maturation is a significant factor (17, 28). However, we did not find significance in other expected covariates. Serum creatinine was not included in the final model as most enrolled patients had normal serum creatinine values over a tight range, which were highly correlated with PNA. We could only enroll one premature neonate under 14 days of age (see Table 1, Cohort 4); thus, we had a limited ability to assess the impact of GA at birth. Similarly, postmenstrual age was also not included in the final model as it is driven by the PNA component of postmenstrual age. In summary, we found that the same dose of oxacillin could be used in infants with normal serum creatinine, whether term or premature (>14 days of age); 160 mg/kg/day continuous infusions of oxacillin will reliably achieve stringent PD targets of *f*T 100% > MIC up to an MIC of 0.5 mcg/mL.

There are many limitations to this study. First, from a feasibility standpoint, clinicians must take into consideration medication incompatibilities that arise with the use of continuous infusions of oxacillin in the setting of limited intravenous access. This can make it difficult to care for infants with serious infections where continuous infusion is beneficial, yet it makes other aspects of clinical care difficult due to limited venous access points for other crucial medications. We frequently encountered medication incompatibilities with concomitant medications and oxacillin being administered simultaneously through the same venous catheter. These included lipid infusions for total parenteral nutrition, sedative medications, and other antimicrobials, which were common medication incompatibilities that we encountered. We lack high-quality data to guide optimal PD targets in neonates, but because prior literature suggests a stringent target of *f*T 100% > MIC as an optimal target for critically ill patients, and considering that *S. aureus* late-onset sepsis is tied to a high mortality, we chose a stringent PD target for our study (29). The number of participants was adequate to describe the overall pharmacokinetics of oxacillin and the impact of PNA on CL but too small to evaluate other potential PK covariates. We were only able to enroll one premature neonate <32 weeks gestational age and <14 days of postnatal age, and thus the model is limited in giving reliable dosing estimates in premature infants under 14 days of age. Serum creatinine, a marker of renal function, is likely a covariate of CL. However, our evaluation was limited because in our enrollees, creatinine exceeded 0.4 mg/dL in mostly infants less than 14 days (which may partially reflect maternal creatinine rather than neonatal clearance) and was tightly distributed in infants over 14 days of age, and only reported to one significant digit with a lower limit of detection of 0.2 mg/dL. We were unable to enroll critically ill patients, so the model may not be applicable to critically ill infants. The sparse sampling due to constraints on phlebotomy in this fragile population and employment of a continuous infusion dosing scheme impaired estimation of Vd and its inter-individual variability. However, collecting our first sample between 30 and 120 minutes after the loading dose provided sufficient information on the typical value of Vd. We also did not measure free-drug concentrations, instead assuming 10% unbound drug concentrations based on literature estimates of oxacillin protein binding (9). This may underestimate the true free-drug concentration in this population: literature on other antimicrobials suggests that protein binding in neonates, particularly premature infants, is likely statistically lower than that observed in adults, which can increase the free-drug concentration and increase the likelihood of achieving PD targets at MICs higher than we observed in our study (30, 31–33). Additionally, a significantly higher blood volume (fourfold greater) would have been demanded for accurate free-drug concentration measurements. Our PK sampling only supported the use of a one-compartment model, and we avoided very early PK sampling that would have been necessary to describe the potential distributive phase and two-compartment model. This resulted in our model underestimating a few extremely high plasma concentrations of oxacillin (i.e., peak concentrations), as evidenced by the goodness of fit plot. However, as oxacillin is a time-dependent antibiotic with efficacy tied to *f*T > MIC, precise predictions of peak concentrations are less important than those later in the dose interval or obtained hours after oxacillin administration, which this model does predict adequately. We also did not perform an external validation of our population PK model, but this was not feasible given the lack of other existing pharmacokinetic literature on oxacillin in the neonatal population; this would be a future area of potential study. The strength of this study is the evaluation of oxacillin exposure using a continuous infusion scheme, which provided useful information on the safety of this regimen and the achievement of steady-state concentrations above MIC targets while on continuous infusion.

In summary, we evaluated the exposure of oxacillin in premature and term neonates and young infants at higher doses than currently recommended. For a confirmed *S. aureus* infection causing neonatal late-onset sepsis, administration of oxacillin as a continuous infusion is safe and preferable to an intermittent infusion, for an MIC up to 0.5, in an infant with normal serum creatinine. As the CLSI breakpoint MIC is 2, institutions that rely on oxacillin for treatment of *S. aureus* infections may need to review their local antibiogram and determine their respective MIC_90_. Perhaps higher doses of oxacillin are more likely to achieve desired PD targets associated with clinical success, but we did not evaluate them with Monte Carlo simulations and we did not evaluate the safety of higher doses in this age group. However, because our data may have underestimated oxacillin concentrations, and true unbound oxacillin concentrations may be higher than anticipated, no definitive conclusion on the likelihood of treatment success can be made for the treatment of MSSA infection with an MIC of 2 with the described continuous infusion regimen. We believe further research is needed to objectively evaluate oxacillin protein binding in this age group with variable serum albumin levels.

## Data Availability

Data is available for review as supplemental material.

## SUPPLEMENTAL MATERIAL

Table S1 aac.01777-25-s0001.xlsx Adverse events listed.

Table S2 aac.01777-25-s0002.xlsx Raw data.

Table S3 aac.01777-25-s0003.xlsx Raw concentrations.

ASM does not own the copyrights to Supplemental Material that may be linked to, or accessed through, an article. The authors have granted ASM a non-exclusive, world-wide license to publish the Supplemental Material files. Please contact the corresponding author directly for reuse.
